# Second primary malignancies of eye and ocular adnexa after a first primary elsewhere in the body

**DOI:** 10.1007/s00417-020-04896-1

**Published:** 2020-09-01

**Authors:** Ahmad Samir Alfaar, Anas M. Saad, Mahmoud Tawfik KhalafAllah, Omneya Ezzat Elsherif, Moataz Hamed Osman, Olaf Strauß

**Affiliations:** 1 Experimental Ophthalmology, Department of Ophthalmology, Charité – Universitätsmedizin Berlin, a corporate member of Freie Universität, the Berlin Institute of Health, Humboldt-University, 13353 Berlin, Germany; 2 Ophthalmology Department, Faculty of Medicine, Leipzig University, Liebigstr, 10-14, 04103 Leipzig, Germany; 3 Cleveland Clinic Foundation, Cleveland, OH USA; 4 Vision Science Graduate Program, University of Alabama, Birmingham, AL USA; 5 Department of Ophthalmology, Menoufia University, Shebin El-Kom, Egypt; 6 Family Medicine, Cairo University Hospitals, Cairo, Egypt; 7 Ophthalmology Department, Kasr Alainy Hospital, Faculty of Medicine, Cairo University, Cairo, Egypt

**Keywords:** Orbit, Eye, Second malignancies, Epidemiology, Ocular adnexa

## Abstract

**Purpose:**

The eye and its adnexal structures can give rise to first or consecutive primary malignancies or to encounter metastasis. Our aim was to define the characteristics of the second primary neoplasms affecting the eye and its adnexa and find the risk modifying factors for them after malignancies elsewhere in the body.

**Methods:**

We have queried the Surveillance, Epidemiology and End-Results “SEER”-9 program of the National Cancer Institute for the malignancies of the eye and its adnexa that occurred between 1973 and 2015. The malignancies were ordered chronologically according to their incidence: first or second primary malignancies. The tumors were classified according to ICD-O-3 classification. Standardized incidence ratios (SIR) and survival probabilities were calculated for subgroups.

**Results:**

Among 3,578,950 cancer patients, 1203 experienced a second malignancies of the eye and its adnexa. The first malignancy was diagnosed between 50 and 69 years of age in 58.94% of them. The eyelid showed 280 events, while 50 in lacrimal gland, 181 in the orbit, 21 in the overlapping lesions, 15 in optic nerve, 148 in the conjunctiva, 9 in the cornea, 6 in the Retina, 379 in the choroid, and 93 in the ciliary body. The SIR of a second malignancy after a prior non-Hodgkin lymphoma was 2.42, and in case of previous skin carcinomas it was 3.02, melanoma of skin, and 2.13 and 1.58 in oral cavity/pharynx malignancies. The second ocular and adnexal neoplasms increased steadily over the 5-year periods on contrary to first primary neoplasms. The survival of patients affected with first ocular and adnexal neoplasms was significantly higher than those with second ocular and adnexal neoplasms. On the other side, second primary ocular and adnexal tumors showed a better survival than second primary malignancies elsewhere.

**Conclusions:**

The epidemiological differences between first and second ocular and adnexal primaries suggest different underlying mechanisms. Careful ocular examination should be integrated in the long-term follow-up plan of cancer patients. Special attention should be given to patients with non-Hodgkin’s lymphoma and melanoma as first primary.
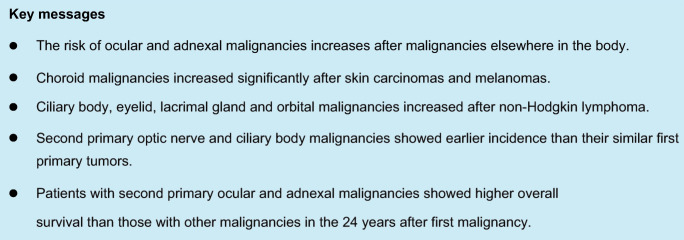

**Electronic supplementary material:**

The online version of this article (10.1007/s00417-020-04896-1) contains supplementary material, which is available to authorized users.

## Introduction

The eye and its ocular adnexa (EOA) are affected by a broad spectrum of malignant tumors. Enhanced by the more prolonged survival of cancer cured patients and the better diagnostic techniques, a rising incidence of second ocular and adnexal primary cancers have been detected. However, the paucity of large case series braked establishing distinct epidemiological patterns [1].

Second primary malignant tumors (SPMTs) are defined as new primary malignant tumors that are encountered after another primary one. Different factors are involved, yet, without an established pattern. Genetic predisposition, environmental factors, and various treatment options are correlated with the second primary malignancies. SPMTs are challenging in many aspects. For diagnosis, symptoms can be overlooked in the context of fatigability attributed to the primary tumor and/or its therapy. For therapy, planning another therapeutic course, like radiotherapy, after a prior one can have various drawbacks, including induction of another malignancy [2–5]**.**

Virtually every malignancy has the potential to send secondaries to the eye and its adnexa. The most common primary sites to metastasize this region are the breast and the lung. In most situations, metastatic tumors at the EOA present first and incite the search for a primary. Breast is an exception, with nearly 90% of its secondaries are discovered after a primary malignancy therapy [6–11].

On the contrary, SPMTs in the ocular and its adnexal region do not have an established epidemiological pattern. The EOA–SPMTs are rarely reported as a distinct entity with calculated risk as other regions. Instead, they are usually categorized with other sites as “others.” Therefore, this study is conducted [2, 5, 12].

In this study, we aimed to explore the major themes of SPMTs in the ocular and its adnexal regionusing data from the Surveillance, Epidemiology and End Results (SEER) Program of the US National Cancer Institute (NCI). Besides, we aimed to delineate how age, gender, and race may impact the risk of the SPMTs.

## Methods

### Study design and data source

This study represents a retrospective cohort study of patients registered in Surveillance, Epidemiology and End-Results “SEER9” registry, which cover about 9.4% of the general US population between 1973 and 2015 [13].

### Study population

We examined the records of patients diagnosed with a second primary cancer in the EOA after being diagnosed with at least one primary prior cancer elsewhere in the body. We allowed at least 2 months period between the diagnosis of the first and the second cancers. We defined cancers of interest as malignancies occurring in the eyelid, conjunctiva, cornea, retina, choroid, ciliary body, lacrimal glands, overlapping lesions of the eye and adnexa, and optic nerve. For this selection, we used the ICD-O-3 topographic classification with the codes: C44.1, C69.0-9, and C72.3. SEER data are anonymized and considered non-human subject research. Thus, it is IRB approval and HIPAA is exempted.

Within all included patients, we have revised the demographical characteristics besides the site of the first and further malignancies, histological subtype of the malignancies using adolescent and young adults (AYA) recode variable, treatment modalities for the first malignancy, vital status at the end of follow up (end of 2015), and the survival period.

### Outcomes and statistical analyses

We have used Multiple Primary-Standard Incidence Ratio (MP-SIR) session in SEER*Stat version 8.3.5 [13] to calculate O/E ratios, which represent the number of actually diagnosed cases of the malignancies of the EA following a prior primary malignancy, divided by the number of expected cases of similar locations in a demographically similar population. To calculate these rates, patients with unknown race were grouped with whites. We calculated O/E ratios for each site of EA cancers, and according to different demographic and tumor-related characteristics.

We performed Kaplan–Meier test to calculate the overall survival of patients who developed second primary EA malignancies. We used the log-rank test to compare the overall survival of these patients with patients who have primary EA malignancies without prior malignancies, and with the overall survival of patients who developed second primary malignancies in sites elsewhere. We used IBM SPSS 24 for survival analyses. All tests were two-sided, and a *p* value that is less than 0.05 was considered statistically significant.

## Results

### Baseline characteristics

**Fig. 1:**
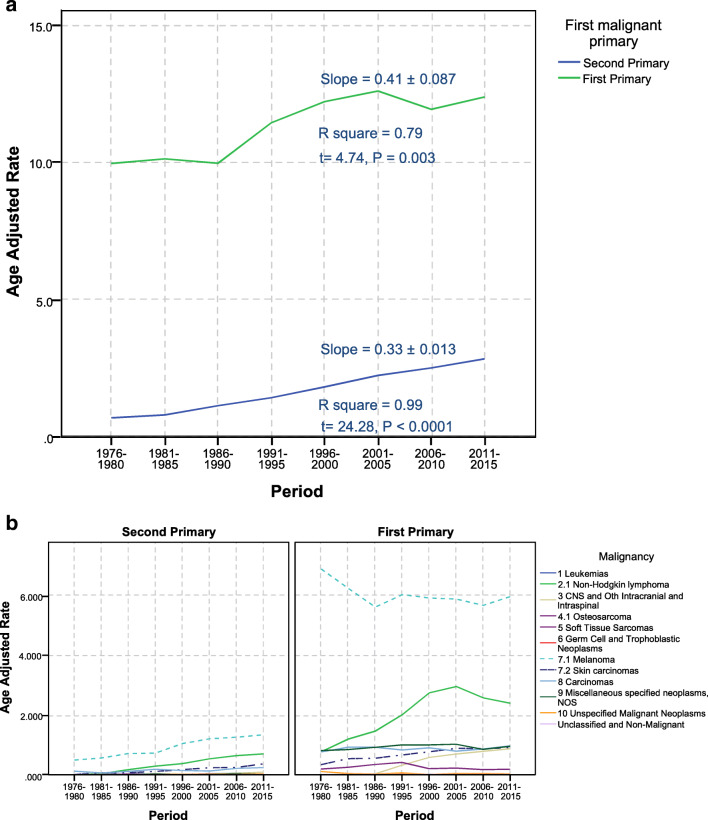
Incidence of the first primary vs. second primary ocular and its adnexal tumors over years. **a** Age adjusted rate for first and second primary. Linear regression details were presented on each line. **b** Age-adjusted rate per AYA major cancer groups

We reviewed a total of 3,578,950 cancer patients’ records, of which 1222 primary malignancies of the EA in 1203 patients after a previous malignancy elsewhere. From those patients, 93.4% were whites, 67% were married, and about 58.94% of them were 50–69 years old when they were diagnosed with their first malignancy. Male-to-female ratio was 1.22, where males represented 54.9% of the patients. The median time to develop a second primary ocular and ocular adnexal malignancy was 9.7 years following the first cancer diagnosis. The most common sites of the first malignancy were prostate and genitourinary tract (24.5%), and breast (17.12%) (Table 1). The age-adjusted rate showed a steady increase over 5-year periods since 1976 (Fig. 1).

**Table 1:** Baseline characteristics of patients at their first malignancy (*n* = 1203)

Characteristics		Number	Column *N* %
Sex	Female	543	45.14%
	Male	660	54.86%
Age group	0–19 years	7	0.58%
	20–39 years	55	4.57%
	40–59 years	333	27.68%
	50–69 years	709	58.94%
	70 years–older	99	8.23%
Race (White, Black, other)	White	1124	93.43%
	Black	34	2.83%
	Other^a^	45	3.74%
Marital status at diagnosis	Divorced	59	4.90%
	Married (including common law)	806	67.00%
	Separated	11	0.91%
	Single (never married)	110	9.14%
	Unknown	87	7.23%
	Widowed	130	10.81%
State	California	232	19.29%
	Connecticut	187	15.54%
	Georgia	71	5.90%
	Hawaii	42	3.49%
	Iowa	169	14.05%
	Michigan	185	15.38%
	New Mexico	54	4.49%
	Utah	79	6.57%
	Washington	184	15.30%
AYA site/WHO 2008^b^	1.2 Acute myeloid leukemia	3	0.25%
	1.3 Chronic myeloid leukemia	4	0.33%
	1.4 Other and unspecified leukemia	19	1.58%
	2.1 Non-Hodgkin lymphoma	83	6.90%
	2.2 Hodgkin lymphoma	4	0.33%
	3.1.1 Specified low-grade astrocytic tumors	1	0.08%
	3.1.3 Astrocytoma, NOS	2	0.17%
	3.2 Other glioma	2	0.17%
	4.1 Osteosarcoma	1	0.08%
	4.2 Chondrosarcoma	3	0.25%
	5.1 Fibromatous neoplasms	1	0.08%
	5.3.1.1 Specified (excluding Kaposi sarcoma)	8	0.67%
	5.3.1.2 Kaposi sarcoma	2	0.17%
	6.1 Germ cell and trophoblastic neoplasms of gonads	9	0.75%
	7.1 Melanoma	114	9.48%
	7.2 Skin carcinomas	6	0.50%
	8.1 Thyroid carcinoma	23	1.91%
	8.2.1 Nasopharyngeal carcinoma	1	0.08%
	8.2.2 Other sites in lip, oral cavity, and pharynx	37	3.08%
	8.2.3 Nasal cav, mid ear, sinus, larynx, ill-def head/neck	19	1.58%
	8.3 Carcinoma of trachea, bronchus, and lung	39	3.24%
	8.4 Carcinoma of breast	206	17.12%
	8.5.1 Carcinoma of kidney	17	1.41%
	8.5.2 Carcinoma of bladder	65	5.40%
	8.5.3 Carcinoma of gonads	14	1.16%
	8.5.4 Carcinoma of cervix and uterus	51	4.24%
	8.5.5 Carc of oth and ill-def sites, geniourinary tract	295	24.52%
	8.6.1 Carcinoma of colon and rectum	131	10.89%
	8.6.2 Carcinoma of stomach	7	0.58%
	8.6.3 Carcinoma of liver and intrahepatic bile ducts	3	0.25%
	8.6.5 Carc oth and ill-def sites, gastrointestinal tract	7	0.58%
	8.7.2 Carcinoma of other and ill-defined sites, NOS	9	0.75%
	9.2.3 Myeloma, mast cell, misc. lymphoreticular neo, NOS	9	0.75%
	9.2.4 Other specified neoplasms, NOS	5	0.42%
	10 Unspecified Malignant Neoplasms	3	0.25%

^a^Includes Asians or Pacific Islanders, and Indian Americans/ Alaska natives

^b^The classification scheme for tumors of adolescents and young adults (AYA)

### Risk of second primary eye or ocular adnexal malignancy following a cancer diagnosis

We found the overall risk of developing a second primary EOA malignancy to increase significantly following a cancer diagnosis (O/E = 1.16, 95%CI (1.09–1.22)). This increase was significant in both males and females (O/E = 1.14, 95%CI (1.06–1.23), and O/E = 1.18, 95%CI (1.08–1.28), respectively). A prior non-Hodgkin lymphoma, or skin melanoma, were associated with significant increases in the risk; O/E = 2.42, 95%CI (1.93–2.99), and O/E = 2.13, 95%CI (1.76–2.55), respectively. On the other hand, a prior colorectal, breast, prostate, respiratory, or urinary bladder cancers, were not associated with significant overall changes in risk.

We further divided the second ocular and adnexal malignancies according to their specific sites. The risks of 2nd primary in the eyelid, ciliary body, and optic nerve malignancies were significantly higher; O/E = 1.35, 95%CI (1.20–1.52), O/E = 1.43, 95%CI (1.16–1.76), and O/E = 3.82, 95%CI (2.14–6.30), respectively. Details on risks of each site are described in Tables 2 and 3.

**Table 2:** Risk of developing second primary malignancy in conjunctiva, cornea, retina, choroid, and ciliary body

	C69.0-Conjunctiva		C69.1-Cornea		C69.2-Retina		C69.3-Choroid		C69.4-Ciliary body		C69.9-Eye, NOS	
	NE^1^	O/E^2^	NE	O/E	NE	O/E	NE	O/E	NE	O/E	NE	O/E
Total	148	1.05	9	0.80	6	0.79	379	1.04	93	1.43#	49	0.98
Sex												
Female	50	1.04	1	0.50	5	1.36	173	1.08	41	1.39	19	0.85
Male	98	1.05	8	0.86	1	0.25	206	1.02	52	1.47#	30	1.09
Age at diagnosis of first malignancy												
0–19 years	0	0.00	0	0.00	1	2.78	0	0.00	1	6.77	0	0.00
20–39 years	6	1.37	0	0.00	0	0.00	18	1.24	7	3.00#	1	0.78
40–59 years	38	1.13	0	0.00	1	0.47	125	1.09	28	1.50	15	1.25
60–79 years	95	1.07	6	0.83	4	0.89	216	1.02	50	1.27	28	0.91
80+ years	9	0.64	3	1.36	0	0.00	20	0.92	7	1.68	5	0.86
Site recode ICD-O-3/WHO 2008 of first malignancy												
All solid tumors	125	0.95	7	0.66	6	0.86	358	1.06	84	1.40#	42	0.91
Oral cavity and pharynx	8	2.30	0	0.00	0	0.00	8	0.88	2	1.17	2	1.63
Digestive system	17	0.81	2	1.04	0	0.00	48	0.96	15	1.57	8	1.02
Respiratory system	10	1.37	1	1.62	0	0.00	11	0.58	7	1.93	4	1.52
Bones and joints	0	0.00	0	0.00	1	79.34#	0	0.00	0	0.00	0	0.00
Soft tissue including heart	0	0.00	0	0.00	0	0.00	2	1.02	1	2.89	0	0.00
Skin excluding basal and squamous	12	1.58	2	3.79	0	0.00	36	1.64#	4	1.12	1	0.40
Melanoma of the skin	11	1.56	1	2.06	0	0.00	35	1.71#	4	1.19	1	0.43
Other non-epithelial skin	1	1.80	1	23.66	0	0.00	1	0.71	0	0.00	0	0.00
Breast	16	0.77	0	0.00	2	1.33	86	1.23	15	1.20	7	0.74
Female genital system	3	0.36	0	0.00	2	3.05	22	0.77	8	1.48	3	0.75
Male genital system	46	1.01	3	0.67	1	0.57	108	1.14	19	1.19	15	1.15
Prostate	44	0.99	3	0.68	1	0.58	103	1.13	19	1.23	15	1.18
Urinary system	9	0.65	0	0.00	0	0.00	26	0.80	10	1.69	2	0.44
Brain and other nervous system	0	0.00	0	0.00	0	0.00	0	0.00	2	8.04	0	0.00
Endocrine system	4	1.58	0	0.00	0	0.00	12	1.50	1	0.76	0	0.00
Thyroid	4	1.65	0	0.00	0	0.00	11	1.43	1	0.79	0	0.00
All lymphatic and hematopoietic diseases	19	2.17#	1	1.54	0	0.00	19	0.80	9	2.15	5	1.64
Lymphoma	15	2.84#	1	2.74	0	0.00	8	0.55	8	3.16#	4	2.22
Hodgkin lymphoma	0	0.00	0	0.00	0	0.00	0	0.00	0	0.00	1	4.64
Non-Hodgkin lymphoma	15	3.26#	1	3.06	0	0.00	8	0.64	8	3.69#	3	1.89
Myeloma	0	0.00	0	0.00	0	0.00	2	0.90	0	0.00	0	0.00
Leukemia	4	1.53	0	0.00	0	0.00	9	1.32	1	0.81	1	1.07
Lymphocytic leukemia	2	0.95	0	0.00	0	0.00	8	1.49	1	1.02	0	0.00
Non-lymphocytic leukemia	2	3.94	0	0.00	0	0.00	1	0.69	0	0.00	1	5.54
Acute non-lymphocytic leukemia	0	0.00	0	0.00	0	0.00	1	1.68	0	0.00	0	0.00
Myeloid and monocytic leukemia	2	4.47	0	0.00	0	0.00	1	0.78	0	0.00	1	6.35
Kaposi sarcoma	1	5.54	0	0.00	0	0.00	0	0.00	0	0.00	0	0.00
Miscellaneous	3	4.41	0	0.00	0	0.00	1	0.55	0	0.00	2	7.59

*NOS* non-otherwise specified

^1^Number of cancer patients who had a second eye/orbit malignancy

^2^The observed over expected ratio

^3^Using AYA site recode/WHO 2008 classification

# *P* < 0.05

**Table 3:** Risk of developing second primary malignancy in eyelid, lacrimal gland, orbit, optic nerve, or overlapping lesions

	C44.1-Eyelid		C69.5-Lacrimal gland		C69.6-Orbit, NOS		C69.8-Overlapping lesion of eye and adnexa		C72.3-Optic nerve	
	NE^1^	O/E^2^	NE	O/E	NE	O/E	NE	O/E	NE	O/E
Total	280	1.35#	50	1.21	181	1.15	21	1.37	15	3.82#
Sex										
Female	134	1.37#	24	1.11	87	1.13	10	1.58	10	6.05#
Male	146	1.34#	26	1.31	94	1.16	11	1.22	5	2.20
Age at diagnosis of second primary										
0–19 years	1	5.78	0	0.00	0	0.00	0	0.00	4	11.51#
20–39 years	12	2.99#	3	1.86	6	1.78	0	0.00	4	7.47#
40–59 years	60	1.39#	18	1.38	45	1.24	5	1.45	3	2.02
60–79 years	171	1.29#	23	0.98	117	1.19	15	1.54	3	2.31
80+ years	36	1.35	6	1.93	13	0.66	1	0.57	1	3.77
Site recode ICD-O-3/WHO 2008 of first malignancy										
All solid tumors	251	1.30#	36	0.94	148	1.01	19	1.33	13	3.75#
Oral cavity and pharynx	12	2.71#	2	2.13	5	1.46	1	2.59	0	0.00
Digestive system	32	1.00	5	0.85	23	0.94	2	0.83	1	2.19
Respiratory system	12	1.22	3	1.47	12	1.59	1	1.17	0	0.00
Bones and joints	1	4.75	0	0.00	0	0.00	0	0.00	0	0.00
Soft tissue including heart	2	1.99	0	0.00	0	0.00	0	0.00	2	38.49#
Skin excluding basal and squamous	53	5.17#	0	0.00	11	1.42	2	2.69	1	3.41
Melanoma of the skin	51	5.39#	0	0.00	9	1.26	2	2.90	1	3.66
Other non-epithelial skin	2	2.55	0	0.00	2	3.34	0	0.00	0	0.00
Breast	47	1.12	8	0.85	27	0.82	6	2.23	2	3.35
Female genital system	17	1.02	1	0.27	11	0.84	1	0.87	0	0.00
Male genital system	49	0.90	12	1.24	44	1.10	6	1.41	3	3.10
Prostate	46	0.86	11	1.17	42	1.07	6	1.45	3	3.40
Urinary system	24	1.30	4	1.20	14	1.02	0	0.00	0	0.00
Brain and other nervous system	0	0.00	0	0.00	0	0.00	0	0.00	3	33.96#
Endocrine system	4	1.17	1	1.03	2	0.72	0	0.00	1	5.69
Thyroid	4	1.22	1	1.07	2	0.75	0	0.00	1	6.49
All Lymphatic and hematopoietic diseases	22	1.80#	14	5.41#	29	3.08#	2	2.18	2	4.73
Lymphoma	18	2.44#	12	7.48#	22	3.87#	0	0.00	1	4.50
Hodgkin lymphoma	0	0.00	2	9.08#	1	1.70	0	0.00	0	0.00
Non-Hodgkin lymphoma	18	2.71#	10	7.23#	21	4.12#	0	0.00	1	6.57
Myeloma	0	0.00	0	0.00	4	4.25#	0	0.00	0	0.00
Leukemia	4	1.10	2	2.80	3	1.08	2	7.10	1	5.61
Lymphocytic leukemia	2	0.67	2	3.59	2	0.89	2	8.73#	0	0.00
Non-lymphocytic leukemia	2	3.03	0	0.00	1	1.91	0	0.00	1	26.54
Acute non-lymphocytic leukemia	2	8.30#	0	0.00	1	5.01	0	0.00	0	0.00
Myeloid and monocytic leukemia	2	3.48	0	0.00	1	2.19	0	0.00	1	29.46
Kaposi sarcoma	0	0.00	0	0.00	1	6.26	0	0.00	0	0.00
Miscellaneous	5	5.14#	0	0.00	2	2.66	0	0.00	0	0.00

*NOS* non-otherwise specified

^1^NE Number of cancer patients who had a second eye/orbit malignancy

^2^O/E The observed over expected ratio

^3^Using AYA site recode/WHO 2008 classification

#*P* < 0.05

On the other hand, 454,005 patients developed second non-EOA primary malignancy after another non-EOA malignancies with a smaller but significant increase in risk; O/E = 1.07, 95%CI (1.07–1.08), but the excess risk was 11.94 per 10,000. Table 1 shows the baseline characteristics of patients who developed an SPMT in the EOA.

To study the impact of having a prior malignancy on the survival of the EOA malignancies, we compared the overall survival of the patients divided according the milestone of where they developed EOA malignancy versus the patients who did develop non-EOA malignancy. Patients with single EOA malignancies with no other associations (*n* = 9376 patients) showed lower survival until 10 years after the diagnosis then showed a better survival after 25 years. On the other side, second EOA malignancies showed steady higher overall survival until 25 years after the diagnosis (median 201 months, 95%CI (188.14–213.86)).

**Fig. 2:**
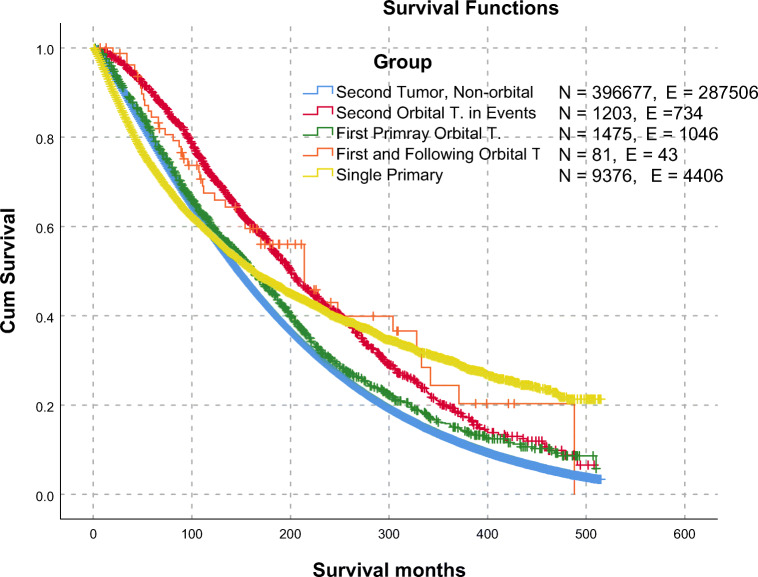
Overall survival comparison between single primary EOA malignant tumors, first primary EOA malignancies (followed by second tumors), second primary EOA tumors after malignancies elsewhere. Legend Details: - Second Tumor, Non-orbital: Patients with a second malignancy, No EOA tumors in first or following events.- Second Orbital T. in Events: Patients with a second malignancy, The second malignancy is an EOA tumor. - First and Following Orbital T.: Patients with first and second primary EOA tumors. - Single Primary: Patients with a single event of EOA malignancies, no second primary tumors. Comparison details are in supplementary Table 2. EOA eye and its adnexa

Patients who developed first and second malignancies both not involving EOA (*n* = 396,677 patients) showed the worst survival 10 years after diagnosis (median 162 months, 95%CI [151.2–172.8) (Fig. 2).

## Discussion

Innocent ocular symptoms, including change of refractive error, dryness, or allergy, may masquerade a hidden malignancy. Moreover, such lesions can be the first sign of metastasis [14, 15]. Secondary malignancy is terminology-wise mistaken with second-primary tumors [16]. Secondary tumors may refer to metastatic tumors or relapsing tumors of ocular or adnexal origin. Our paper focuses on the risk of developing a second malignancy in the eye or its adnexa after the incidence of a previous primary malignancy elsewhere in the body.

Improvements in the early detection and the treatment protocols have led to more cancer survivors, hence an increase in the number of patients developing subsequent cancers. We found that the overall risk of developing a second primary EOA malignancy increases significantly following a cancer diagnosis compared with the incidence of the primary EOA malignancies in general population. This is consistent with many other studies that documented the incidence of second malignancies among cancer survivors. Understanding the pattern of second malignancies is essential for planning the follow-up and screening protocols after the first cancer diagnosis.

Curtis et al. found that cancer survivors had a 14% higher risk to develop a new malignancy compared with that expected in the general population. They showed cumulative incidence of 5.0%, 8.4%, 10.8%, and 13.7% at 5, 10, 15, and 25 years, respectively [17]. This risk may be a result of the lifestyle, genetic predisposition, environmental exposures, and/or the effects of cancer therapy [18].

Many studies evaluated the risk of a second malignancy in specific sites. Between 1973 and 2011, Laíns et al. followed patients diagnosed with uveal melanoma as their first malignancy. They found an 11% excess risk of a second malignancy, mainly due to a significantly increased risk of skin melanomas and kidney tumors. Radiotherapy showed no effect on this risk [18]. Abramson et al. reported 28 third tumors developed in 211-s tumor survivors of retinoblastoma patients within a median time of 5.8 years [19].

The pattern of second primary malignancies in our results contradicts that in metastatic tumors where the breast followed by the lung were the most common primary sites for metastasis either for the ocular adnexa or the eye [11, 20, 21]**.**

In our study, SIR was statistically significant in females and those between 20 and 39, and 40–59 years. Among the sites of primary cancers, the skin melanoma came first, followed by non-Hodgkin’s lymphoma (NHL). This is in agreement with different studies that did not report SPMT in the EOA region after breast cancer [4, 22, 23] and prostate [24]**.**

Age pattern for second malignancies in the EOA is different from that for second malignancies elsewhere, where our results showed 58.9% of the patients developing second malignancies in the EOA are in the age range of 50–69 years at their first presentation. While the previously published relative risk of developing second malignancies was 6-fold higher for survivors of childhood cancer. This may be attributed to the effect of initial therapy, genetic susceptibility, and the age effect [17]. Both males and females showed a significantly higher risk of a second malignancy in the EOA, which is consistent with Youlden et al. [16]. Curtis et al. reported that females had a slightly higher relative risk than males for all subsequent cancers [17].

Regarding the type of first primary tumor, we found that prior NHL was associated with a significant increase in the risk of second malignancies in the lacrimal gland, ciliary body, orbit, and eyelid. NHL is associated with SPMT in different sites. In a large-scale analysis of NHL cases, the EOA region was reported to develop SPMT with a SIR of 1.73. This is in agreement with our results, while the difference in the SIR may be attributed to the combined analysis of the eye and the ocular adnexal regions in their analysis [3]. Similarly, NHL had a pooled RR of 1.43 for SPMT in the EOA region in a meta-analysis, which included 23 studies [5]. However, the combined analysis for the whole region may account for the lower SIR in these studies compared with ours. Nonetheless, the excess risk for SPMT after NHL is well documented and reported [25, 26].

Eyeball malignancies are not far from the orbital ones, sharing similar challenges. Ocular metastasis is well studied, being the most common intraocular malignancy, where near half of these tumors originate in the breast, followed by lung and prostate [6, 20]. SEER central quality control paid attention to differentiate between metastasis and second primaries according to IARC Guidelines. Therefore, the observed patterns in our study are different from the distribution of other common malignancies and their metastasis.

Conjunctiva is another site that possessed a significantly higher risk of SPMT after NHL with no significant influence of gender or age. Eyelid is a known site for primary as well as metastatic tumors. As well, it showed a higher risk for SPMT that was significantly higher for males, females, and all age groups in our analysis. Basal and squamous cell carcinomas were the most prevalent primary eyelid malignancies. Similarly, they were significantly detected as SPMT in the eyelid. Skin melanoma and NHL were reported as primary malignancies for SPMT in the eyelid. As well, they may be the SPMT in the eyelid in contrast to their rare primary occurrence in the eyelid [27–29]. The paucity of the literature of eyelid SPMT hinders comparing our results to explore matching or discrepancy.

Lacrimal gland tumors are common among the white race with an age-adjusted incidence rate of 0.6 per 1,000,000. In the USA, NHL is the most common primary lacrimal gland malignancy. In our study, the lacrimal gland did not show a significantly higher overall risk for SPMT. NHL dominated as the only significant pathological type of SPMT. Nonetheless, the lacrimal gland was not reported among SPMT after NHL elsewhere [3, 5]. Lacrimal gland secretion decreases with radioiodine in thyroid carcinoma therapy, a mechanism that may contribute to the risk of SPMT in the lacrimal gland after radiation for other malignancies [30]**.**

EOA tumors are among the most challenging malignancies. Five-year survival after the exenteration for orbital tumors reached 57% [31]. SPMTs in the EOA are not an exception for this, our study showed a significantly worse survival in the former.

Figure 1 shows different patterns in annual incidence rates of first and second primaries where first primary malignancies showed multiphasic pattern and second primary malignancies showed a steady increase. The potential causes for first primary malignancies were discussed elsewhere [32]. Our paper presents a new pattern in second ocular and its adnexal malignancies that may not be influenced by the same factors. Such point is a potential for future further research for investigating the underlying molecular and genetic differences between the cancers especially in non-Hodgkin’s lymphoma and melanoma.

Our literature review of the most common pathological subtypes and/or sites revealed shared predisposing genes (Supplementary Table 1). We believe that such shared genetic factors may play a role for predicting the incidence of a second malignancy. Moreover, it can guide minimizing the panel used for targeting the second malignancy.

The current study has superior reliability compared with other case studies in terms of the primary sites reported and the histological type of malignancies. However, the retrospective nature of our study carries a potential risk of bias due to the misreporting of the primary malignancy for SPMT or other demographic data. Other limitations are related to the coding of cancer registries, where some anatomical regions are not represented in topographical classifications, e.g., orbital bones and minute orbital structures, e.g., ganglia.

In conclusion, we propose that second ocular and its adnexal primary malignancies are driven by a different underlying mechanism from first primaries. Moreover, a primary malignancy should raise the suspicion for screening either for metastasis or SPMT with any innocent presentation. So, it is recommended to perform a thorough ophthalmic examination with particular attention to the eyelid, the conjunctiva, and the eyeball in the follow-up of cancer cured patients. This is of paramount importance if the first primary malignancy was after the age of 20 years in white race patients. As well, a prior history of any of NHL, EOA malignancy, melanoma, and oropharyngeal malignancies should be thoroughly assessed for potential SPMT in the EOA region.

## Electronic supplementary material

Supplementary Table1. Genetic mutations shared between first primary and second primary malignancies affecting eye and ocular adnexa . (DOCX 33.9 kb)

Supplementary table 2: Kaplan- Meier, Overall Survival, Log-Rank Pairwise Comparison Details for Figure 2. (DOC 143 kb)

## Data Availability

Not applicable.
