# Supplementary material for: Second primary malignancies of eye and ocular adnexa after a first primary elsewhere in the body
Source: Graefes Arch Clin Exp Ophthalmol. 2020 Sep 1;259(2):515–26. doi: 10.1007/s00417-020-04896-1 (PMC7843581; doi:10.1007/s00417-020-04896-1)
Supplement: Supplementary file 1 — Genetic mutations shared between first primary and second primary malignancies affecting eye and ocular adnexa . (DOCX 33.9 kb) [file 417_2020_4896_MOESM1_ESM.docx]

Supplementary Table1. Genetic mutations shared between first primary and second eye and its adnexal primary malignancies.

| Gene | Sk. Mel.  ^1^ | Osteos.  ^2^ | DLBCL  ^3^ | HL  ^4,5^ | ALL  ^6^ | AML  ^7^ | Br. Ca  ^8^ | CLL  ^9^ | CRC  ^10,11^ | OrPh Ca  ^12^ | Pros. Ca  ^13,14^ | UB Ca  ^15^ | Freq. |
| --- | --- | --- | --- | --- | --- | --- | --- | --- | --- | --- | --- | --- | --- |
| TP53 | x | x | x | x |  | x | x | x | x | x |  | x | 10 |
| PTEN | x | x |  |  |  |  | x |  | x | x | x |  | 6 |
| MYC |  | x | x | x |  |  |  | x |  |  | x |  | 5 |
| CDKN2A | x | x | x |  |  |  |  |  |  | x |  | x | 5 |
| NOTCH1 |  |  | x |  | x |  |  | x |  | x |  |  | 4 |
| RB1 |  | x |  |  |  |  |  | x |  |  |  | x | 3 |
| BRAF | x |  |  |  |  |  |  | x | x |  |  |  | 3 |
| MLH1 |  | x |  |  |  |  | x |  | x |  |  |  | 3 |
| ATM |  | x |  |  |  |  | x | x |  |  |  |  | 3 |
| EGFR |  | x |  |  |  |  | x |  |  | x |  |  | 3 |
| CDH1 |  |  |  |  |  |  | x |  |  | x |  |  | 2 |
| ARID1A |  | x | x |  |  |  |  |  |  |  |  |  | 2 |
| BCR-ABL1 |  |  |  |  | x | x |  |  |  |  |  |  | 2 |
| B2M |  |  | x | x |  |  |  |  |  |  |  |  | 2 |
| BRCA1 |  | x |  |  |  |  |  |  |  |  | x |  | 2 |
| REL |  |  |  | x |  |  |  | x |  |  |  |  | 2 |
| MSH2 |  |  |  |  |  |  | x |  | x |  |  |  | 2 |
| RUNX1 |  | x |  |  |  | x |  |  |  |  |  |  | 2 |
| NFKBIE |  |  |  | x |  |  |  | x |  |  |  |  | 2 |
| TERT | x |  |  |  |  |  |  |  |  |  |  | x | 2 |
| PIK3CA |  | x |  |  |  |  |  |  |  | x |  |  | 2 |
| HRAS |  |  |  |  |  |  |  |  |  | x |  | x | 2 |
| BRCA2 |  | x |  |  |  |  |  |  |  |  | x |  | 2 |
| XPO1 |  |  |  | x |  |  |  | x |  |  |  |  | 2 |
| BAP1 | x | x |  |  |  |  |  |  |  |  |  |  | 2 |
| STK11/LKB1 |  | |  |  |  |  | x |  | x |  |  |  | 2 |
| EZH2 |  |  | x |  |  |  |  |  |  |  | x |  | 2 |
| TET2 |  |  | x |  |  | x |  |  |  |  |  |  | 2 |
| GATA3 |  |  |  | x | x |  |  |  |  |  |  |  | 2 |
| AKT1 |  | x |  |  |  |  |  |  |  |  |  | x | 2 |

Acronyms; SK. MEL.: Skin Melanoma, OSTEOS.: Osteosarcoma, DLBCL: Diffuse large B-cell lymphoma, HL: Hodgkin lymphoma, ALL: Acute lymphoblastic leukemia, AML: BR. CA.: Breast Cancer, CLL: Chronic lymphocytic leukemia, CRC: Colorectal cancer, ORPH CA: Oropharyngeal carcinoma, PROS. CA: Prostate carcinoma , UB CA: Urinary bladder carcinoma, FREQ.: Frequency

References:

1. Cheng L, Lopez-Beltran A, Massari F, MacLennan GT, Montironi R. Molecular testing for BRAF mutations to inform melanoma treatment decisions: a move toward precision medicine. *Mod Pathol*. 2018;31(1):24-38. doi:10.1038/modpathol.2017.104

2. Rickel K, Fang F, Tao J. Molecular genetics of osteosarcoma. *Bone*. 2017;102:69-79. doi:10.1016/j.bone.2016.10.017

3. Pasqualucci L, Dalla-Favera R. Genetics of diffuse large B-cell lymphoma. *Blood*. 2018;131(21):2307-2319. doi:10.1182/blood-2017-11-764332

4. Steidl C, Telenius A, Shah SP, et al. Genome-wide copy number analysis of Hodgkin Reed-Sternberg cells identifies recurrent imbalances with correlations to treatment outcome. *Blood*. 2010;116(3):418-427. doi:10.1182/blood-2009-12-257345

5. Borchmann S, Engert A. The genetics of Hodgkin lymphoma: an overview and clinical implications. *Curr Opin Oncol*. 2017;29(5):307-314. doi:10.1097/CCO.0000000000000396

6. Tasian SK, Hunger SP. Genomic characterization of paediatric acute lymphoblastic leukaemia: an opportunity for precision medicine therapeutics. *Br J Haematol*. 2017;176(6):867-882. doi:10.1111/bjh.14474

7. Bullinger L, Döhner K, Döhner H. Genomics of Acute Myeloid Leukemia Diagnosis and Pathways. *J Clin Oncol*. 2017;35(9):934-946. doi:10.1200/JCO.2016.71.2208

8. Banerji S, Cibulskis K, Rangel-Escareno C, et al. Sequence analysis of mutations and translocations across breast cancer subtypes. *Nature*. 2012;486(7403):405-409. doi:10.1038/nature11154

9. Ghamlouch H, Nguyen-Khac F, Bernard OA. Chronic lymphocytic leukaemia genomics and the precision medicine era. *Br J Haematol*. 2017;178(6):852-870. doi:10.1111/bjh.14719

10. DE ROSA M, PACE U, REGA D, et al. Genetics, diagnosis and management of colorectal cancer (Review). *Oncol Rep*. 2015;34(3):1087-1096. doi:10.3892/or.2015.4108

11. Munteanu I, Mastalier B. Genetics of colorectal cancer. *J Med Life*. 7(4):507-511.

12. Tan M, Myers JN, Agrawal N. Oral Cavity and Oropharyngeal Squamous Cell Carcinoma Genomics. *Otolaryngol Clin North Am*. 2013;46(4):545-566. doi:10.1016/j.otc.2013.04.001

13. Shen MM, Abate-Shen C. Molecular genetics of prostate cancer: new prospects for old challenges. *Genes Dev*. 2010;24(18):1967-2000. doi:10.1101/gad.1965810

14. Karan D, Lin M-F, Johansson SL, Batra SK. Current status of the molecular genetics of human prostatic adenocarcinomas. *Int J Cancer*. 2003;103(3):285-293. doi:10.1002/ijc.10813

15. Zhang X, Zhang Y. Bladder Cancer and Genetic Mutations. *Cell Biochem Biophys*. 2015;73(1):65-69. doi:10.1007/s12013-015-0574-z
