# Supplementary material for: Second primary malignancies of eye and ocular adnexa after a first primary elsewhere in the body
Source: Graefes Arch Clin Exp Ophthalmol. 2020 Sep 1;259(2):515–26. doi: 10.1007/s00417-020-04896-1 (PMC7843581; doi:10.1007/s00417-020-04896-1)
Supplement: Supplementary file 2 — Kaplan- Meier, Overall Survival, Log-Rank Pairwise Comparison Details for Figure 2. (DOC 143 kb) [file 417_2020_4896_MOESM2_ESM.doc]

Supplementary data 2: Kaplan- Meier, Overall Survival, Log-Rank Pairwise Comparison Details for Figure 2

Case Processing Summary	
Group	Total N	N of Events	Censored	
			N	Percent	
Not Index Not Event	396677	287506	109171	27.5%	
Event Only	1203	734	469	39.0%	
Index Only	1475	1046	429	29.1%	
Index and Event	81	43	38	46.9%	
Single Primary	9376	4406	4970	53.0%	
Overall	408812	293735	115077	28.1%	


Means and Medians for Survival Time	
Group	Meana	Median	
	Estimate	Std. Error	95% Confidence Interval	Estimate	Std. Error	95% Confidence Interval	
			Lower Bound	Upper Bound			Lower Bound	Upper Bound	
Not Index Not Event	180.713	.245	180.233	181.193	148.000	.292	147.428	148.572	
Event Only	227.926	4.878	218.364	237.487	201.000	6.563	188.137	213.863	
Index Only	195.167	4.303	186.734	203.600	162.000	5.520	151.181	172.819	
Index and Event	240.829	21.954	197.799	283.858	214.000	30.057	155.089	272.911	
Single Primary	228.909	2.670	223.675	234.142	162.000	4.024	154.113	169.887	
Overall	181.419	.244	180.941	181.897	148.000	.290	147.432	148.568	

a. Estimation is limited to the largest survival time if it is censored.	


Pairwise Comparisons	
	Group	Not Index Not Event	Event Only	Index Only	Index and Event			
		Chi-Square	Sig.	Chi-Square	Sig.	Chi-Square	Sig.	Chi-Square	Sig.			
Log Rank (Mantel-Cox)	Not Index Not Event			98.899	.000	11.066	.001	8.060	.005			
	Event Only	98.899	.000			30.734	.000	.164	.686			
	Index Only	11.066	.001	30.734	.000			4.451	.035			
	Index and Event	8.060	.005	.164	.686	4.451	.035					
	Single Primary	164.524	.000	8.114	.004	15.699	.000	1.371	.242			
